# Cytokines in Bipolar Disorder: Paving the Way for Neuroprogression

**DOI:** 10.1155/2014/360481

**Published:** 2014-09-09

**Authors:** Izabela Guimarães Barbosa, Moisés Evandro Bauer, Rodrigo Machado-Vieira, Antonio Lucio Teixeira

**Affiliations:** ^1^Divisão de Neuropsiquiatria, Laboratório Interdisciplinar de Investigação Médica, Faculdade de Medicina, Universidade Federal de Minas Gerais (UFMG), Avenida Alfredo Balena, 190 Santa Efigênia, 30130-100 Belo Horizonte, MG, Brazil; ^2^Laboratório de Imunologia do Envelhecimento, Instituto de Pesquisas Biomédicas, Pontifícia Universidade Católica do Rio Grande do Sul (PUC-RS), 90610-000 Porto Alegre, RS, Brazil; ^3^Laboratório de Neurociências, LIM27, Instituto e Departamento de Psiquiatria, Universidade de Sao Paulo, 01060-970 Sao Paulo, SP, Brazil; ^4^Experimental Therapeutics and Pathophysiology Branch, National Institute of Mental Health, National Institutes of Health, Bethesda, MD 20852, USA; ^5^Instituto de Estudos Avançados Transdisciplinares (IEAT), UFMG, 31270-901 Belo Horizonte, MG, Brazil

## Abstract

Bipolar disorder (BD) is a severe, chronic, and recurrent psychiatric illness. It has been associated with high prevalence of medical comorbidities and cognitive impairment. Its neurobiology is not completely understood, but recent evidence has shown a wide range of immune changes. Cytokines are proteins involved in the regulation and the orchestration of the immune response. We performed a review on the involvement of cytokines in BD. We also discuss the cytokines involvement in the neuroprogression of BD. It has been demonstrated that increased expression of cytokines in the central nervous system in postmortem studies is in line with the elevated circulating levels of proinflammatory cytokines in BD patients. The proinflammatory profile and the immune imbalance in BD might be regarded as potential targets to the development of new therapeutic strategies.

## 1. Introduction

Bipolar disorder (BD) is a chronic, severe, and disabling medical illness. The lifetime prevalence is estimated at 2.4% [1]. The cardinal diagnostic feature is the occurrence of at least one episode of mania and/or hypomania, although depressive episodes tend to predominate in the course of the illness. The mean age at onset is around 20 years old. The risk of mood episodes remains constant after 40 years of the illness, indicating a risk of recurrence of symptoms until around 70 years of age [2]. BD patients present significant mood symptoms during at least half of their lifetime [3]. The cyclic nature of manic and depressive symptoms has been appointed as the major cause of disability in BD patients [4].

BD is characterized by a temporal progression of symptoms, that is, increase in the frequency and severity of mood episodes and less response to treatment [5–8]. Several studies have also reported cognitive impairment along with structural (decrease of hippocampal and amygdala volumes and total gray matter) and neurophysiological changes [5–9]. The term “neuroprogression” was applied to refer to this temporal clinical progression in BD based on the notion of clinical staging used in oncology and internal medicine. One of the main factors associated with the neuroprogression and, consequently, with the prognosis is the frequency of mood episodes (mania or depression). Hence the higher the frequency of mood episodes is associated with the fastest neuroprogression changes and the compromised prognosis. The biological mechanisms underlying BD neuroprogression are not determined and may involve complex interactions among multiple genes and environmental factors leading to impairment in several physiological systems [5].

Indeed BD has been regarded as a multisystemic condition, impairing cognitive, endocrine, autonomic, and circadian rhythms. There is an elevated incidence of psychiatric disorders like anxiety disorders, obsessive compulsive disorder, alcohol and substance abuse, attention-deficit/hyperactive disorder, and eating disorders [1, 10]. BD is frequently comorbid with several medical conditions, including cardiovascular and metabolic diseases (particularly* diabetes mellitus* and obesity) that partially contribute to the reduced lifetime expectancy in these patients [11, 12]. The cooccurrence of autoimmune diseases has also been described. For instance, case-control studies showed that BD patients present high frequency of systemic lupus erythematosus [13], multiple sclerosis [14, 15], and autoimmune thyroiditis [16]. Recently a cohort study showed that a history of Guillain-Barre syndrome, Crohn's disease, or autoimmune hepatitis was associated with a raised risk of BD [17].

A growing body of evidence, represented mainly by the finding of increased circulating levels of proinflammatory cytokines, suggests that immune-mediated mechanisms are related to the neurobiology of BD and its neuroprogression. Cytokines, a broad category of small proteins, are traditionally involved in the orchestration of immune responses [18]. Besides this classical role, they can directly affect neuronal activity, inducing neuronal excitability and plastic changes [19]. Moreover cytokines can influence the hypothalamic-pituitary-axis (HPA) through effects on the HPA feedback regulation and on the glucocorticoid receptor function [20]. Cytokines activate the HPA axis, increasing the levels of corticotrophin releasing hormone (CRH), adrenocorticotropic hormone (ACTH), and cortisol and decreasing the expression, translocation, and downstream effects of glucocorticoid receptors [21]. The resulting net effect is a persistent elevation of glucocorticoids levels which has been consistently associated with mood symptoms [22]. Cytokines may also interfere with the metabolism of neurotransmitters, such as serotonin and dopamine, in determined brain regions (amygdala, hippocampus, and* nucleus accumbens*) involved in the regulation of emotion, reward, and psychomotor functions [23, 24]. Indeed both proinflammatory cytokines can stimulate the enzyme indoleamine 2,3-dioxygenase (IDO) [25]. IDO converts tryptophan into kynurenine (KYN), reducing the availability of this precursor of serotonin [22–26]. Proinflammatory cytokines also enhance the activity of the enzyme kynurenine-3-monooxygenase enzyme (KMO) that degrades KYN into 3-hydroxykynurenine, shifting the KYN pathway into the production of neurotoxic metabolites.

In the present paper, we performed a comprehensive review on the role of cytokines in BD, also addressing their involvement in illness progression. The following search strategy was applied: online search of the databases MEDLINE and SCOPUS from 1990 until May 2014 using the keywords (MESH criteria): “bipolar disorder” AND “cytokines.” Only papers published in English were evaluated.

## 2. Evidence of Altered Cytokines in Bipolar Disorder

### 2.1. Evidence of Cytokine-Related Genes in Bipolar Disorder

BD is strongly influenced by genetic factors. The concordance in monozygotic twins is 67% and the concordance in dizygotic twins is 19%, and the estimate heritability is around 80% [27]. Among several candidate genes, some studies investigated the association between BD and immune-related genes polymorphisms.

Two case-control studies reported that BD is associated with an uncommon variant with an adenine (A) at position −308 of the tumor necrosis factor alpha (TNF-*α*) gene which is related with higher TNF-*α* production [28, 29]. However, two other case-control studies failed to confirm such association [30, 31].

Regarding the interleukin- (IL-) 1 family, four case-control studies evaluated its expression and polymorphisms in BD. In BD patients, the expression of a genetic variant of the IL-1*β* gene, the variant (−511T), was associated with volume decrease of the gray matter, especially in the left dorsolateral prefrontal cortex, indicating a role for proinflammatory mechanisms in brain structural changes [32]. Two studies demonstrated that the presence of a variable number of tandem repeats (VNTR) in intron 2 of the IL-1Ra gene (IL1RN) (IL1RN *2) confers susceptibility to BD [33], particularly in BD patients with a positive family history of the illness [34]. Nevertheless, a third study failed to confirm this finding [35]. Interestingly, IL1RN *2 allele was associated with more prolonged and more severe proinflammatory immune response when compared with other IL1RN genotypes in humans.* In vitro *studies also demonstrated that the presence of the IL1RN *2 allele was associated with enhanced IL-1*β* production and decreased production of IL-1Ra [36, 37].

Three case-control studies revealed that BD patients present a significant association between the uncommon variant with an adenine (A) at position −2581 of the CCL2 gene that is associated with higher production of the chemokine CCL2/MCP-1 (involved in the recruitment of monocytes, memory T cells and dendritic cells into inflammatory sites) under proinflammatory stimulus [38–40]. Despite the reported associations between BD and immune-related genes, none of them has been confirmed by meta-analyses including genome-wide association studies [41, 42].

Regarding transcriptomic changes, Padmos et al. (2008) [43] reported that monocytes from BD patients and their offspring had overexpression of mRNA cytokines, notably TNF-*α*, IL-1*β*, IL-6, and CCL2. One hypothesis is that the aberrant RNA processing of cytokines in BD might be determined by (not yet defined) epigenetic mechanisms influencing not only cytokines levels but also the neurobiology of the illness.

### 2.2. Neuroimaging and Postmortem Studies and Their Relationship with Cytokines in Bipolar Disorder

There are still a limited number of studies evaluating brain abnormalities and cytokines in BD patients at the same time point. Hsu et al. [44] found that serum levels of IL-10 correlated with serotonin transporter availability (as assessed by single-photon emission computed tomography (SPECT)) in the thalamus, indicating a possible link between peripheral inflammation and monoamine metabolism in the brain. Conversely, one study failed to find any association between peripheral IL-1 receptor antagonist (IL-ra) levels and white matter integrity [45].

*Postmortem studies* demonstrated the presence of dendritic atrophy of neurons and the loss of oligodendroglial cells in BD, notably in the medial prefrontal cortex [46]. Only few studies have assessed immune markers in this context, revealing increased inflammatory and decreased anti-inflammatory markers in the frontal cortex of BD patients compared with controls [47, 48]. More specifically, they reported increased protein and mRNA levels of IL-1*β* and its receptor (IL-1R), NF-*κ*B transcription factor subunits (p50 and p65), and astroglial and microglial markers (glial fibrillary acidic protein, inducible nitric oxide synthase, c-fos and CD11b) [47]. Conversely, BD patients showed decreased total RNA expression of transforming growth factor (TGF) *β* in the frontal cortex [47]. Dean et al. [49] also showed that BD patients present increased transmembrane TNF-*α* protein level in the anterior cingulate area and decreased TNF-*α* receptor 2 protein levels in the dorsolateral prefrontal cortex in comparison with controls. Since the dorsolateral prefrontal cortex is known to be critical in executive functioning, the anterior cingulate in mood control and all these regions are implicated in BD [50]; these data suggest a role for cytokines in disease pathogenesis and also in mood and cognition modulation. Nevertheless, studies assessing whether there is any association between neuropathological findings and clinical (cognitive or psychopathological) parameters are lacking.

Taking into account that microglia is one of the major sources of cytokines in the central nervous system (CNS), these cells were investigated in BD.* Postmortem* studies reported decreased number and size of microglia in BD patients [51]. To conciliate these contradictory results, it was hypothesized that persistent microglia activation in early stages is associated with cellular death in the long term due to failure in the control of anabolic and catabolic cellular machineries, corroborating the view of a neurodegenerative process in BD [52].

### 2.3. Circulating Cytokine Levels in Bipolar Disorder

Four meta-analyses evaluated peripheral levels of cytokines in BD patients in comparison with controls [53–56]. The concentrations of IL-4, TNF-*α*, sTNFR1, and sIL-2R were significantly higher in BD patients in comparison with controls [53–56]. There were no significant differences between BD patients and controls for IL-1*β*, IL-2, IL-5, IL-6, IL-8, IL-12, IFN-*γ*, TGF-*β*1, IL-1ra, and sTNFR2 [53–56]. A meta-analysis assessing specifically chemokines did not find any association with BD [56].

As mood state seems to influence the expression of cytokines, findings according to different mood states are shown below.

#### 2.3.1. Euthymia

Euthymia has been associated with a mild proinflammatory profile represented mainly by increased sTNFR1 levels (Table 1). Two studies showed that BD patients in euthymia presented increased CXCL10 plasma levels [57, 58]. This finding might suggest a Th1 skewed immune response in euthymia as the chemokine CXCL10 is a potent attractant of activated Th1 cells. Only one study investigated cytokines in the cerebrospinal fluid of BD patients, reporting increased levels of IL-1*β* [59].

#### 2.3.2. Mania and Depression

The immune changes already observed in euthymia are enhanced during mania (Table 2) and depressive state (Table 3). BD patients in mania exhibited increased circulating levels of IL-6, TNF-*α*, sTNFR1, IL-ra, and also CXCL10, CXCL11, and IL-4. Besides the association with inflammatory molecules, mania was associated with Th1 (increased CXCL10 [58]) and Th2-type cytokines (increased CXCL11 [60] and IL-4 [48, 59, 61]). BD patients in depression had increased circulating levels of sTNFR1 and CXCL10. It is worth mentioning that the number of studies assessing peripheral cytokines in depressive BD is much lower than mania or euthymia.

#### 2.3.3. Circulating Cytokine Levels in Bipolar Disorder Compared with Other Psychiatric Disorders

There are few studies comparing BD with other psychiatric disorders, and it is not possible to draw a definite conclusion on specific immune markers for BD. Regardless the mood state, BD patients did not differ from subjects with schizophrenia on peripheral levels sTNFR1, IL1ra, IL-6, IL-10, and IL-12 [62–65] or from major depression patients when assessing TNF-*α*, IL-6, and IL-12 [64, 66–68]. One study found that BD patients during a mood episode (mania or depression) had decreased IL-1*β* levels in comparison with major depression patients [69]. These findings limit the potential use of cytokines and other inflammatory mediators as diagnostic biomarkers for BD.

### 2.4. Production of* In Vitro* Cytokines by Peripheral Mononuclear Blood Cells

In whole blood assays, Kim et al. [70] showed increased levels of IL-6 and TNF-*α* following different stimuli (phytohemagglutinin and lipopolysaccharide), but no changes in the production of IL-2, IL-4, and IFN-*γ* in BD patients. Studies evaluating the release of cytokines by stimulated lymphocytes reported lower production of IFN-*γ* [60, 65] in BD patients compared with controls, with no difference in IL-4 [69] and IL1-*β* [71]. Data regarding the production of IL-6 [67, 68], IL-2 [67, 68], and IL-10 [60, 68, 72] are contradictory, with some studies showing decreased production of IL-2, IL-6, and IL-10 [72], while others report no significant differences [60, 68, 71].

It seems an apparent paradox the finding of chronic low-grade inflammation in BD, but no response (or even decrease) in cytokines production under immune stimuli* in vitro*. We hypothesize that the immune system is already overstimulated in BD patients [73], not being able to respond to additional stimuli.

## 3. Cytokines and Their Relationship with Neuroprogression and Comorbidities in Bipolar Disorder Patients

BD has been associated with increased peripheral levels of proinflammatory cytokines, and this mild chronic inflammation tends to exacerbate during mood episodes. The frequency of mood episodes (mania or depression) is inversely associated with BD outcome as the higher their frequency, the worst the disease prognosis. Therefore, mood episodes seem to play a pivotal role in BD progression, and one of the putative mechanisms would be enhanced inflammatory response during mania or bipolar depression [5]. In this scenario, proinflammatory cytokines would act as major “toxic players,” contributing to psychopathological changes, cognitive impairment, and related comorbidities and could be regarded as potential biomarkers for neuroprogression in BD [74–76]. However the studies assessing stage-related cytokines changes were cross-sectional, and longitudinal or prospective evidence for this cytokine effect is lacking. For instance, Kauer-Sant'Anna et al. [77] found that the proinflammatory cytokines IL-6 and TNF-*α* were elevated in both early- (<3 years of disease) and late-stage (>10 years of disease) BD, whereas the anti-inflammatory cytokine IL-10 was increased only in the early stage. Another study described elevated levels of proinflammatory cytokines only in patients with long-duration BD compared with short-duration disease [78]. These preliminary findings suggest that there is a tendency of enhanced inflammation with disease progression.

To date evidence of structural and neuropsychological changes derived, respectively, from neuroimaging and clinical studies supports the concept of staging in BD. Longer lifetime illness duration was related to lower total gray matter, even when controlling for age [7]. Number of manic episodes is associated with cognitive impairment, specifically deficits in executive function, episodic memory, and reduced psychomotor slowing [79].

The neurobiological processes underlying neuroprogression are still undetermined, but inflammatory mechanisms seem to play a major role. TNF-*α* acts on TNFR1 being capable of inducing neuronal cell death through the activation of caspases and apoptotic machinery [80, 81], TNF-mediated process might contribute to the volumetric reduction, and hypoactivation of frontal lobes in BD [82, 83] which are associated with disinhibition of limbic structures [84]. This corticolimbic dysfunction may underlie the emotional dysregulation and cognitive impairment associated with BD. In line with this, our and other groups have consistently demonstrated that there is a positive correlation between increased proinflammatory cytokines levels and cognitive impairment, particularly involving frontal lobe functions [45, 85, 86].

The increased proinflammatory state and the elevated rate of medical comorbidity in BD patients are also associated [11]. One of the possible pathways is the persistent activation of the HPA axis. Medical conditions, including cardiovascular and endocrine diseases, can cause and are exacerbated by chronic stress and HPA axis activation [83, 84, 87–89]. HPA axis activity presents a major role in the regulation of immune response. Otherwise inflammatory cytokines may stimulate HPA axis activity. Hence mood episodes can trigger stress response-related mechanisms, leading to hyperactivation of the HPA axis and enhanced proinflammatory status contributing to the development and/or exacerbation of the comorbid medical conditions in BD. The medical conditions* per se* cannot explain the whole proinflammatory state in BD patients, as differences in relation to controls persist even when controlling for confounding factors. Recently we demonstrated that overweight BD patients had increased proinflammatory profile when compared with overweight controls, corroborating this assumption [62].

## 4. Perspectives

Current pharmacological therapeutics for BD are based on neurotransmitters dysfunction. The available strategies are quite effective in relieving behavioral and psychological symptoms mainly related with mania and preventing relapses, but the management of BD depression remains a great challenge [90]. Moreover, the available drugs do not target and can even worsen medical comorbidities associated with BD such as obesity and insulin resistance [91].

It is worth mentioning that the mechanisms underlying the therapeutic effect of mood stabilizers and other drugs used in BD are not fully understood. There is preclinical and clinical evidence on these drugs modulating cytokines and playing a role in proinflammatory pathways. Valproic acid decreases the stimulated* in vitro *proinflammatory cytokines production in healthy controls [92], while lithium, antipsychotics, and antidepressants also inhibit proinflammatory cytokines production and/or synthesis in animal and* in vitro *studies [93, 94].

The development of new therapeutic targets in the treatment of BD is highly needed and the immune system is a promising target. In this scenario, anti-inflammatory strategies derived from four major classes, namely, polyunsaturated fatty acids (PUFAs), cyclooxygenase (COX) inhibitors, anti-TNF, and minocycline have been tested in BD patients [95]. In a seminal study, Nery et al. [96] conducted a double blind, randomized, placebo-controlled add-on study within depressive or mixed episodes of BD patients using an COX 2 inhibitor (celecoxib). In this study the treatment with celecoxib was associated with a faster decrease in depression scores compared with placebo [96]. This study indicates a potential antidepressant effect of anti-inflammatory medications related with the inflammatory/immune imbalance in these patients [96]. New double-blind controlled studies evaluating other anti-inflammatory drugs (i.e., aspirin and minocycline) in BD patients are underway [95, 97]. However, a word of caution is necessary as the relation between BD and inflammation seems to be much more complex than a mere “pro-inflammatory status,” represented by elevated circulating levels of cytokines. This can be exemplified by the unexpected clinical observation of a series of cases of mania induced by TNF-antagonists (infliximab and etanercept) [98–100].

## 5. Conclusion

BD is a complex and multisystemic condition with an underlying neurobiology. Evidences have appointed increased peripheral and central inflammatory cytokines levels in BD patients. Thus, it is plausible believe that proinflammatory cytokines might contribute to the pathophysiology, clinical comorbidities, and neuroprogression of BD. Future studies should focus on their role as biomarkers of neuroprogression and the evaluation and development of immune-based therapeutic strategies in BD.

## Figures and Tables

**Table 1:** Cytokine peripheral levels in bipolar disorder patients in euthymia compared with controls.

Cytokine	Findings	Conclusion
TNF	*↔* [44, 57, 61, 62, 73, 77, 85, 86, 101–105]	*↔*
	↑ [63, 70]	

sTNFR1	*↔* [73, 106, 107]	↑
	↑ [61, 103, 105, 108]	

sTNFR2	*↔* [61, 73, 103]	*↔*
	↑ [105]	

IFN-*γ*	*↔* [63, 86]	NC
	↑ [69, 70]	

IL-2	*↔* [63, 86]	*↔*
	↑ [70]	

IL-4	*↔* [86, 104]	NC
	↑ [69, 70, 77]	
	↓ [63]	

IL-6	*↔* [45, 57, 77, 86, 101, 102, 104]	*↔*
	↑ [63, 70, 106]	

IL-10	*↔* [48, 86]	NC
	↑ [44, 70]	
	↓ [104]	

IL-17 A	*↔* [86]	NC
	↑ [70]	

IL-1*β*	*↔* [60, 62, 104]	*↔*
	↑ [45, 106]	

IL-1 ra	*↔* [63, 107]	NC
	↑ [45, 109]	

IL33	↑ [58]	↑

sST2	*↔* [58]	*↔*

CCL2	*↔* [45, 59, 85, 104]	*↔*

CCL3	*↔* [59, 73]	*↔*

CXCL8	*↔* [104]	NC
	↓ [59]	

CXCL10	↑ [59, 86]	↑

CXCL11	*↔* [45, 85]	NC
	↑ [59]	

CXCL24	↑ [59]	NC
	↓ [85]	
		

↓: decreased levels; *↔*: not altered levels; ↑: increased levels; NC: not conclusive.

**Table 2:** Cytokine peripheral levels in bipolar disorder patients in mania state compared with controls.

Cytokine	Findings	Conclusion
TNF	*↔* [77]	↑
	↑ [101, 110, 111]	

sTNFR1	*↔* [107]	↑
	↑ [61, 106]	

sTNFR2	*↔* [61]	*↔*

IL-6	↑ [77, 111]	*↔*
	*↔* [45, 62, 64, 101, 106]	

IL-4	↑ [48, 77, 110]	↑

IL-1*β*	*↔* [59, 110]	NC
	↑ [43]	

IL-12	*↔* [110]	↑

IL-1 ra	*↔* [45, 70]	*↔*

IL33	↑ [58]	↑

sST2	*↔* [58]	*↔*

CCL2	*↔* [109]	*↔*

CCL3	*↔* [69]	*↔*

CXCL8	↑ [59]	NC
	↓ [69]	

CXCL10	↑ [69]	↑

CXCL11	↑ [69]	↑

CXCL24	*↔* [69]	*↔*

↓: decreased levels; *↔*: not altered levels; ↑: increased levels; NC: not conclusive.

**Table 3:** Cytokine peripheral levels in bipolar disorder patients in depressive state compared with controls.

Cytokine	Findings	Conclusion
TNF	*↔* [58, 59, 82, 83]	*↔*
	↑ [59, 66, 109]	

sTNFR1	↑ [45]	↑

IL-6	*↔* [45, 59, 61, 66, 111]	*↔*
	↑ [101]	

IL-4	*↔* [101]	NC
	↓ [109]	

IL1-*β*	*↔* [107, 109]	*↔*
	↑ [42]	

IL-1 ra	*↔* [45]	*↔*
	↑ [69]	

↓: decreased levels; *↔*: not altered levels; ↑: increased levels; NC: not conclusive.
